# Adult neurogenesis in the mushroom bodies of red flour beetles (*Tribolium castaneum*, Herbst) is influenced by the olfactory environment

**DOI:** 10.1038/s41598-020-57639-x

**Published:** 2020-01-23

**Authors:** Björn Trebels, Stefan Dippel, Magdalina Schaaf, Karthi Balakrishnan, Ernst A. Wimmer, Joachim Schachtner

**Affiliations:** 1 Philipps-University Marburg, Department of Biology, Animal Physiology, Karl-von-Frisch-Str. 8, 35032 Marburg, Germany; 2 Department of Forest Zoology and Forest Conservation, Georg-August-University Göttingen, Büsgen-Institute, Büsgenweg 3, Göttingen, 37077 Germany; 3 Department of Developmental Biology, Georg-August-University Göttingen, Johann-Friedrich-Blumenbach-Institute for Zoology and Anthropology, GZMB, Ernst-Caspari-Haus, Justus-von-Liebig-Weg 11, Göttingen, 37077 Germany; 4 Clausthal University of Technology, Adolph-Roemer-Str. 2a, 38678 Clausthal-Zellerfeld, Germany

**Keywords:** Adult neurogenesis, Olfactory system

## Abstract

Several studies showed adult persisting neurogenesis in insects, including the red flour beetle *Tribolium castaneum*, while it is absent in honeybees, carpenter ants, and vinegar flies. In our study, we focus on cell proliferation in the adult mushroom bodies of *T. castaneum*. We reliably labelled the progenies of the adult persisting mushroom body neuroblasts and determined the proliferation rate under several olfactory conditions within the first week after adult eclosion. We found at least two phases of Kenyon cell proliferation in the early adult beetle. Our results suggest that the generation of Kenyon cells during the first three days after adult eclosion is mainly genetically predetermined and a continuation of the developmental processes (nature), whereas from day four on proliferation seems to be mainly dependent on the odour environment (nurture). Considering that the mushroom bodies are linked to learning and memory, neurogenesis in the mushroom bodies is part of the remodelling of neuronal circuits leading to the adaption to the environment and optimization of behaviour.

## Introduction

The ground pattern of the central nervous system is genetically encoded. Following development, when the nervous system first encounters environmental sensory input, it is crucial for the survival of an animal to adapt to the actual conditions and cues. Therefore, the genetically encoded wiring scheme of the nervous system must be altered. This plasticity typically occurs in special time windows of elevated susceptibility to sensory input (critical periods or sensitive phases)^1^.

The research on critical periods has mainly focused on vertebrates, where critical periods were among others found during the postnatal development of the visual system^2–10^ and song learning in birds^11–13^. However, several studies demonstrated the existence of critical periods also in insects^14–19^, with many focusing on the plasticity of the olfactory system in the vinegar fly *Drosophila melanogaster*^15,17–22^.

Many insects rely on their olfactory systems to accomplish various tasks, such as the localization of food sources, mating partners, and suitable habitats. The adult olfactory system of holometabolous insects is first confronted with environmental cues upon adult eclosion. These olfactory cues are mainly detected by the olfactory sensory neurons (OSN) housed in the chemosensory sensilla of the antennae and palps^23–26^. From there the information is relayed into the primary olfactory processing centres^27–29^ and further projected to the mushroom bodies (MB) and the lateral horn^26,28–30^, with the former being linked to olfactory learning and memory^31–45^.

Studies in adult holometabolous insects, including *Apis mellifera* and *D. melanogaster*, described synaptic plasticity, but no neurogenesis, in the mushroom bodies^36,46–55^. Since adult neurogenesis in the mushroom bodies was first described in the hemimetabolous house cricket *Acheta domestica*^56,57^, it has also been found in holometabolous insects, such as the noctuid moth *Agrotis ipsilon*^58^, as well as several beetle species^59^ including the red flour beetle *Tribolium castaneum*^60^.

*T. castaneum* is a member of the largest insect order (Coleoptera)^61,62^ and a major pest of stored cereal products^61,63,64^. With its annotated genome sequence^26,65–67^, an expanding genetic toolbox^68–70^ including strong systemic RNA interference (RNAi)^71–73^, and its relative longevity of up to two years^74^, *T. castaneum* represents an eligible beetle model for studying adult plasticity.

We concentrated on olfaction which is supposed to be a main input into the mushroom bodies of *T. castaneum*^26^, to answer whether the adult persisting neurogenesis is genetically predetermined and continuation of developmental processes or if it is activity-dependent.

## Results

### Identification of newly born cells

**Figure 1:**
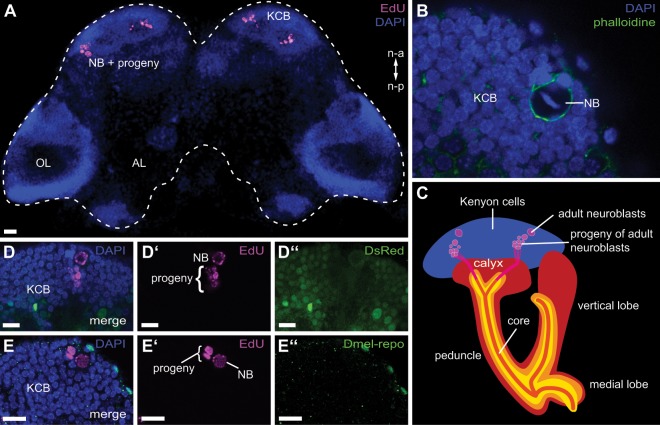
Localization and identification of the adult-born cells as Kenyon cells. (**A**) Volume rendering of an exemplary wholemount preparation showing the location of the adult-born neurons. Nuclei visualized with DAPI are depicted in blue, while the neuroblasts (NB) and their progeny marked by EdU are depicted in magenta. OL: optic lobes; AL: antennal lobe; KCB: cell bodies of Kenyon cells; n-a: neural axis anterior; n-p: neural axis posterior (**B**) Single confocal section showing an MB neuroblast (NB), which can be identified by its larger diameter compared to the surrounding Kenyon cells (KCB). The f-actin of the cytoskeleton is marked by phalloidin (green) and cell nuclei by DAPI (blue). (**C**) Schematic of the adult-born Kenyon cells and their integration into the mushroom bodies. They derive from usually two distinct neuroblasts per hemisphere and send their axons into the core of the mushroom body lobes as shown by Zhao and colleagues^60^.(**D**-D”) Single confocal slice showing colocalization between the EdU marked cells (neuroblasts [NB] and their progeny) and the DsRed reporter signal in the neuron marking transgenic EF1-B-DsRed line^77^. (**E**-E”) Single confocal slice showing EdU marked cells (neuroblasts [NB] and their progeny) and immunohistochemistry against the glia cell marker reversed polarity (anti-repo)^78^ in the San Bernadino wildtype strain. A colocalization cannot be identified.

To label adult-born cells, we used the 5-ethynyl-2′-desoxyuridine (EdU) method^75,76^. With this method, we reliably and exclusively labelled the mushroom body neuroblasts and their progeny, while neurogenesis is not present in other areas of the cerebral ganglion of the red flour beetle (Fig. 1A). The neuroblasts were distinguished from their progeny based on their larger size (Fig. 1B).

We confirmed that in *T. castaneum*, adult-born Kenyon cells usually derive from two neuroblasts (NB) per hemisphere and send their axons into the core of the mushroom body peduncle (PED) and lobes as shown by Zhao and colleagues^60^ (Fig. 1C).

The neuronal identity of the EdU labelled cells was verified by demonstrating co-localization with the reporter signal (Fig. 1D-D”) in the neuron labelling EF1-B-DsRed line^77^, as well as immunohistochemical staining against the glia cell marker reversed polarity^78^ resulting in no co-localization (Fig. 1E-E”).

### Environmental conditions influence adult neurogenesis

**Figure 2:**
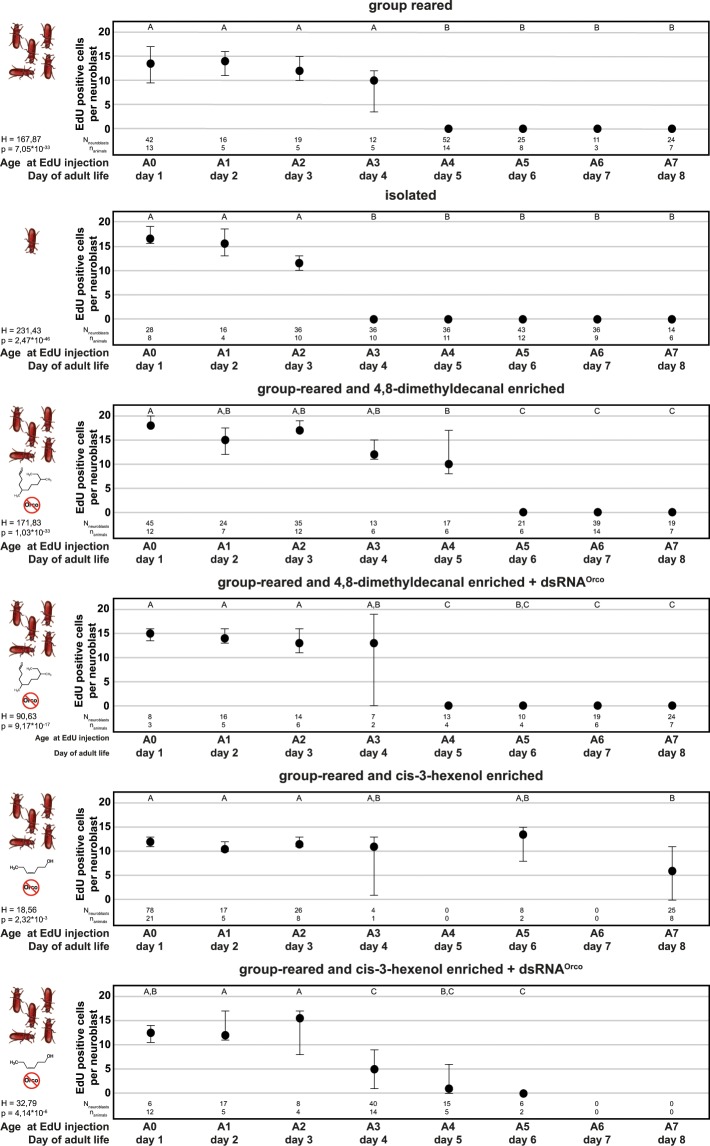
Kenyon cell proliferation in adult beetles under different conditions. Median numbers of EdU positive (adult-born) Kenyon cells per neuroblasts within 24 hours after EdU injection. Sample sizes for each group (analysed neuroblasts [N] and animals[n]) are given in the respective column of the plot. The circles represent the median, while the error bars represent the 95% confidence interval of the median equivalent to the standard error calculated by bootstrap analysis. Kruskal Wallis test with posthoc analysis using Dunns’ multiple comparison test was used to compare the cell number over the different injection times within one experimental group. The results of the Kruskal Wallis test [test statistics corrected for ties (H), p-value (p)] for each condition is given in the figure. Same capital letters indicate groups with no significant difference to each other as obtained by Dunn’s posthoc analysis (p > 0.05, Holm-corrected). The actual p-values of the post-hoc test are listed in Supplementary Table S2. Original photo of the beetle by Peggy Greb, USDA Agricultural Research Service (ID: D268-1).

The proliferation rates of the mushroom body neuroblasts during the first week after adult eclosion were analysed on a day-to-day basis (Fig. 2). A statistical comparison using Kruskal-Wallis test between both sexes showed no major intersexual difference in the number of newly born Kenyon cells, deriving from a single neuroblast (Supplemental Table 1). Thus, for all further analysis, both sexes were pooled. Testing for no variation between conditions and ages by means of Scheirer-Ray-Hare test resulted in rejection of the null hypothesis (olfactory conditions: H = 114.899, p = 0.0; ages; H = 655.154, p = 0.0; overall: H = 231.580, p = 0.0). Further, per condition Kruskal-Wallis analysis between ages showed significant differences between cell numbers at different days, which were further analysed by post-hoc analysis using Dunn’s multiple comparison test (Fig. 2).

For enrichment of the olfactory environment we chose the beetles’ aggregation pheromone 4,8-dimethyldecanal (DMD)^79^ and the food-related green-leaf volatile cis-3-hexenol as both are known to elicit antennal responses in EAG recordings^80^ and at least DMD causing a clear OR/Orco (TcOR1) dependent behavioural response^81^.

The mushroom body neuroblasts of beetles reared in mixed-sex groups of 20 individuals generate new Kenyon cells within the first four days after adult eclosion. During the first three days of the proliferation phase, the median daily proliferation rate of each neuroblast is 10 to 15 cells, with a reduction on day four (Figs. 2, S1). At day 5 the proliferation rate drops significantly and afterwards no neurogenesis is detectable.

Isolating beetles as pupae lead to a proliferation phase lasting three days after adult eclosion. During this proliferation phase, each neuroblast gives rise to about 10 to 20 cells per day, decreasing on day three and significantly dropping at day four before from day five onwards no neurogenesis is detectable.

Enriching the environment of group-reared beetles with DMD leads to a prolonged proliferation phase of five days. During the first three days of the proliferation phase, the median daily proliferation rate of each neuroblast equals to about 15 to 20 cells, with slight decrease on days four and five. At day 6 the proliferation rate drops significantly and afterwards no neurogenesis is detectable.

Stimulating olfactory-deprived (pupal dsRNA^*orco*^ injection) group-reared beetles with DMD leads to a proliferation phase lasting four days after adult eclosion. During the first four days each neuroblast’s median daily proliferation rate is 10 to 15 cells, with a high variation on day four (Figs. 2, S1). At day 5 the proliferation rate drops significantly and afterwards no neurogenesis is detectable.

Stimulation of group-reared beetles with cis-3-hexenol results in mushroom body neuroblasts generating new Kenyon cells during the first six days after adult eclosion with a median rate of 10 to 15 cells per day, decreasing to about 5 cells at day 8.

Stimulating olfactory-deprived (pupal dsRNA^*orco*^ injection) group-reared beetles with cis-3-hexenol leads to a proliferation phase lasting four days after adult eclosion. During the first three days of the proliferation phase, the daily proliferation rate per is 10 to about 15 cells, with a significant reduction on day four (Figs. 2, S1) before dropping to zero at day 6.

### Antennal responses to cis-3-hexenol and 4,8-dimethyldecanal

Electroantennographical recordings (EAG) were used to demonstrate that beetles with an RNAi-mediated systemic knockdown of Orco (dsRNA^*orco*^, test group) differ in their response to the tested odours, compared to beetles injected with sham (DsRed) dsRNA (dsRNA^*sham*^, control group).

**Figure 3:**
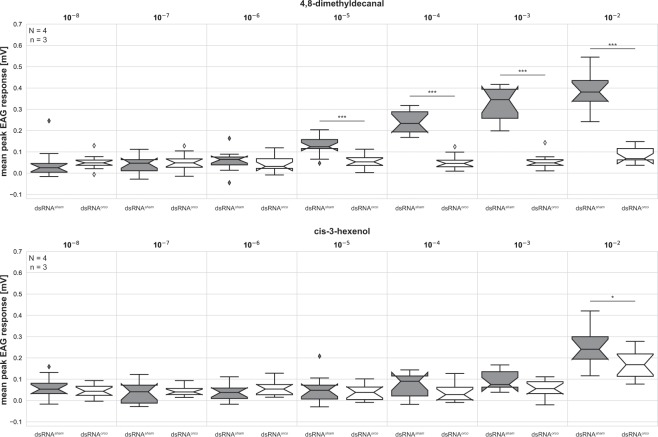
Peak amplitude EAG responses to 4,8-dimethyldecanal and cis-3-hexenol. Box plots with whiskers representing the 5–95% percentile of the peak amplitude EAG response after robust LOESS smoothing and normalization (subtraction of the response to silicone oil, which was used as solvent) to 4,8-dimethyldecanal (DMD) and cis-3-hexenol. Sample sizes are given in the respective subplots. [N] represents the number of animals, while [n] represents the number of replicates per animal. Notches indicate the 95% confidence interval of the median. The bar represents the 25–75 percentile, the line represents the median and the diamonds represent data points outside the 5–95% range. Statistical analysis between dsRNA^*sham*^ and dsRNA^*orco*^ was performed per odour dilution by Kruskal Wallis test. Statistical significance levels of difference in median are represented by asterisks (*p < 0.05, **p < 0.01, ***p < 0.001).

Stimulation with the three lowest tested concentrations (10^−8^ − 10^−6^) of the beetle’s aggregation pheromone DMD leads to no obvious reaction of the antennae (Fig. 3, 4,8-dimethyldecanal; Supplemental Figs. S2 and S3). Starting with a dilution of 10^−5^, a dose-dependent reaction of the antennae is visible in dsRNA^sham^ injected beetles, but not in beetles injected with dsRNA^*orco*^.

Stimulation with the five lowest tested concentrations (10^−8^ − 10^−4^) of the food volatile cis-3-hexenol leads to no obvious responses. Stimulating with a dilution of 10^−3^ causes a slight response in the control group only. Whereas, at the highest tested concentration (10^−2^) both groups respond (Fig. 3, cis-3-hexenol; Supplemental Figs. S2 and S3), but the peak response of the control group is significantly higher compared to the test group (Fig. 3, cis-3-hexenol). Furthermore, the response onset in the *orco* knock-down is delayed (Supplemental Fig. S2).

## Discussion

Neurogenesis in adult brains is present to varying extents throughout invertebrates and vertebrates^57,82–89^. Several studies showed adult neurogenesis in the mushroom bodies of hemimetabolous and ametabolous insects such as crickets^56^, cockroaches^90^, and firebrats^91,92^. In holometabolous insects persisting adult neurogenesis in the mushroom bodies was described in the black cutworm *Agrotis ipsilon*^58^ and several beetles, including the red flour beetle *T. castaneum*^59,60^, while it is absent in the mushroom bodies of the honeybee *A. mellifera*^54^, the carpenter ant *Camponotus floridanus*^34^, and the vinegar fly *D. melanogaster*^55^.

Localization of adult neurogenesis in the mushroom bodies using 5-bromo-2′-deoxyuridine (BrdU) as published for the red flour beetle by Zhao and colleagues^60^ gave no reliable results in our hands and was not useful for the comparison of larger experimental groups needed for the current study. Instead, we successfully and reliably labelled mushroom body neuroblasts and their progeny using the EdU method^75,76^. Like BrdU, EdU is a thymidine analogue that is incorporated into the DNA during replication. The major advantage compared to BrdU is the labelling procedure. While BrdU is localized via immunohistochemistry and therefore requiring the DNA to be denaturized to allow binding of the antibody, EdU is labelled by selective direct chemical coupling with an azide-fluorochrome. Avoiding denaturation of the DNA with HCl provides improved overall tissue preservation. By using this reliable labelling method for newly born neurons and their neuroblasts (Fig. 1) we studied the generation of new Kenyon cells in the early adulthood of the red flour beetle *T. castaneum* under different conditions (Figs. 2, S1) to answer whether this adult neurogenesis depends on olfactory input and if there is a critical period. The activity-dependent remodelling of neuronal circuits during critical periods leads to the adaption to the environment and optimization of behaviour^93^. Previous studies in *A. mellifera* and *D. melanogaster* showed that critical periods during which the mushroom body circuitry is remodelled exist in holometabolous insects and that the underlying mechanisms are the refinement of old as well as the growth of new synapses^48–52,54^, but not neurogenesis^54,55^. The adult olfactory system of the beetle is first confronted with odour cues directly after adult eclosion.

Rearing beetles in groups of 20 animals of both sexes leads to the generation of new Kenyon cells during the first four days after adult eclosion. This phase is shortened in isolated beetles by one day and prolonged by additional olfactory stimulation. Enriching the olfactory environment of group-reared beetles with the beetles’ aggregation pheromone DMD^79^ prolongs the proliferation phase by one day, while stimulation with the food odour cis-3-hexenol leads to a prolongation of the proliferation phase for at least four days. This prolonging effect is mostly inhibited in beetles with a significantly reduced perception of the tested odorants (Fig. 3, dsRNA^*orco*^), generated by RNAi mediated knockdown of *orco*.

A study in crickets already demonstrated that sensory deprivation by isolation results in significantly less neurogenesis among the Kenyon cells of young females, compared to group-reared females^82^. A second study based on these results asked what proportion of neurogenesis is caused by visual and olfactory stimuli^83^ and demonstrated that olfactory and visual deprivation is sufficient to decrease neurogenesis in adult crickets, regardless whether reared in groups or isolation. This clearly showed a link between sensory input and adult neurogenesis.

In our study, we used a day-to-day analysis of adult neurogenesis together with the specific manipulation of the Orco dependent olfactory sensory neurons. This allowed us to temporally dissect adult neurogenesis into distinct phases and link them to olfactory activity.

Similarly to the results from crickets^82^, our data suggest that isolation reduces adult neurogenesis. We were able to demonstrate that this reduction is based on a shortened proliferation phase rather than an altered proliferation rate.

It seems that the generation of Kenyon cells during the first three days after adult eclosion is mainly genetically predetermined and a continuation of developmental processes rather than depending on sensory activity.

The shorter proliferation phase in isolated beetles compared to group-reared beetles suggest an influence of social interactions. This is supported by the fact that the proliferation phase after *orco* knock-down in odour stimulated beetles is still longer than in isolated beetles. Since the beetles were kept under constant darkness, visual stimulation can be excluded, making tactile and gustatory cues the most likely triggers. This is partially in accordance with findings in crickets, were unilateral removal of the antennae, causing the loss of chemosensory as well as mechanosensory antennal input in the ipsilateral hemisphere, lead to less adult neurogenesis in the ipsilateral hemisphere^83^.

The significant differences in the proliferation rate within the first three days might indicate an influence of odour stimulation. However, as the results for DMD and cis-3-hexenol are contradicting, we conclude that these differences are very likely artificial.

However, the main and most striking effect of DMD stimulation is the prolongation of the proliferation phase compared to group-reared beetles, which is not present in dsRNA^*orco*^ injected beetles. Since after a RNAi mediated knockdown of *orco* the EAG response towards DMD is significantly reduced (Fig. 3) and no longer measurable (Supplemental Fig. S2) and the behavioural response towards DMD is abolished^81^, the prolongation is olfactory induced and driven via activity of the OR/Orco complex.

Interestingly, the potential for a longer capacity of the MB system to produce new Kenyon cells seems to depend on the odour, as the stimulation with the food-related odour cis-3-hexenol leads to a longer proliferation phase when compared to DMD stimulation, with cis-3-hexenol causing an increased proliferation rate even at day eight. This seems to contradict the EAG data that suggest a higher sensitivity towards DMD. However, the lack of responses to cis-3-hexenol at lower concentrations does not necessarily mean that it is not perceived, as the recorded response likely depends on the localisation of the OSNs relative to the recording electrode^94^.

This longer proliferation phase is massively shortened in dsRNA^*orco*^ injected beetles, clearly demonstrating that this effect is most likely olfactory but not solely driven via the activity of the OR/Orco complex. While after knockdown of *orco*, the EAG responses to DMD are fully abolished, the EAG responses to cis-3-hexenol show a significantly lower, but still exiting reaction (Fig. 3) with a delayed response onset (Supplemental Fig. S2), which suggests the involvement of another receptor type. As Getahun and colleagues^95^ already described in *D. melanogaster* the best candidates with slower responses are the ionotropic glutamate-like receptors (IR)^26^.

The different effects of DMD and cis-3-hexenol on the duration of the proliferation phase (five vs. eight days) might be explained already on the receptor level, as IRs, unlike ORs, do not exhibit adaptation to longer stimulations^95^. This could mean that continued exposure to high DMD concentration leads to a desensitisation of the OR/Orco complex, causing the shorter proliferation phase. Whereas, the response to cis-3-hexenol seemingly also perceived by IRs persists longer. Furthermore, there is the possibility of different pheromone vs. normal odour processing networks in the antennal lobes that provide the input to the MB as shown among others in *Manduca sexta*^96^, *D. melanogaster*^97^, and *A. mellifera*^98^. However, so far, such separate olfactory processing networks were not described in the red flour beetle and it remains unclear whether they exist.

The EAG responses showed that the reaction of both odorants is significantly reduced in adult female beetles seven days after dsRNA^*orco*^ injection, corresponding roughly to A4. Besides, we performed immunohistochemical staining against Orco in freshly eclosed (A0) and seven days old beetles (A7) using the cross-reactive Moth-R2 antiserum^26^. This resulted in no immunoreactivity after pupal injection of dsRNA^*orco*^ (Supplemental Fig. S4) demonstrating the effectivity of the RNAi within the studied ages. Furthermore, the RNAi effect in *T. castaneum* has already been published to last from weeks to months^99^.

*T. castaneum* has a life span of up to two years^74^ and studies on the origin of the beetle^63^ together with a large variety of odorant receptors^26,81^ suggest a broad spectrum of potential food sources, which may change over time. Adapting the MB neuronal network via newly born cells could be part of the strategy to cope with ongoing environmental changes. The process might be triggered by changes in the OR repertoire in response to the environmental changes, as speculated by Dippel and colleagues^26^. This could explain why Zhao *et al*.^60^. occasionally found neurogenesis in 88 days old beetles without stimulation. The involvement of adult proliferation in behavioural adaption to the changing odorant environment has already been suggested in crickets^82,83^. In analogy, a study on the mouse hippocampus demonstrated that adult neurogenesis is not triggered by continued long-term exposure to enriched environments, but by novel complex stimuli^100^.

Considering that the mushroom bodies are linked to learning and memory^39^, adult neurogenesis might contribute to the formation of new odorant memories. As Zhao and colleagues demonstrated, the adult-born Keyon cells send their axons into the core of the mushroom bodies^60^, which were shown to be involved in differential olfactory learning in *D. melanogaster*^101,102^. Suppression of adult neurogenesis in crickets leads to a significant impairment in olfactory learning and memory^44^, undermining the role of adult-born neurons in learning and memory. Furthermore, studies in vertebrates show that olfactory enrichment leads to increased neurogenesis in the hippocampus and plays a role in olfactory learning^103–106^.

## Methods

### Animals

Red flour beetles (*Tribolium castaneum*, Herbst 1797; Insecta, Coleoptera) of the wild-type strains “San Bernadino”^107^ and *“black”*^108^, as well as the neuron labelling^26,77^ transgenic line EF1-B-DsRed (elongation factor1-alpha regulatory region-DsRedExpress; kindly provided by Michalis Averof, Institut de Génomique Fonctionnelle de Lyon, France) were used. The beetles were bred under constant darkness at about 28 °C and 40–50% relative humidity on organic whole grain wheat flour supplemented with 5% dried yeast powder and 0.05% Fumagilin-B (Medivet Pharmaceuticals Ltd., High River, Alberta, Canada) to prevent sporozoan infections^109^. For age determination, freshly eclosed beetles (A0) were collected and kept in mixed-sex groups of 20 in 68 ml *Drosophila* vials on about 20 g substrate.

For isolation experiments, individuals were separated as pupae into 5 ml glass vials on about 2 g substrate.

### EdU injections

Cold anaesthetized beetles were mounted ventral-up on microscope slides using double-faced adhesive tape and were subsequently injected with about 1 µl 5-ethynyl-2′-desoxyuridine (EdU) solution (100 µM EdU in injection buffer: 1.4 mM NaCl, 0.07 mM Na_2_HPO_4_, 0.03 mM KH_2_PO_4_, 4 mM KCL, and 5% phenol red). Injection was performed with glass micropipettes made from thin-walled glass capillaries (TW150-4, World Precision Instruments, Sarasota, FL, USA; micropipette puller: Sutter Model P-30, Sutter Instrument, Novato, CA, USA) attached to a pressure ejection system (PDES-02T; npi electronics, Tamm, Germany). After injection, the beetles were transferred into a para-film sealed petri dish and incubated at 28 °C for 24 hours.

### Immunohistochemistry and EdU detection

The brains of cold anaesthetized beetles were dissected in PBS (phosphate-buffered saline, 0.01 M, pH 7.4) and fixed in 0.01 M PBS containing 4% paraformaldehyde for 1–2 hours at room temperature or at 4 °C overnight. Fixation was stopped by rinsing 4 × 10 min in PBS supplemented with 0.3% Triton X-100 (PBS-TrX). Afterwards, the brains were transferred into a blocking solution (5% normal goat serum (NGS, Dianova, Hamburg, Germany) in PBS-TrX) and incubated for 3–4 hours at room temperature or overnight at 4 °C. The brains were then incubated with primary antibody solution (PBS-TrX, 2% NGS, concentrations and details see Table 1). After 2–3 days at 4 °C, the antibodies were removed by rinsing 5 × 10 minutes in PBS-TrX. Subsequently, the brains were incubated for 2–3 days at 4 °C in constant darkness with secondary antisera and fluorescent markers (for details see Table 1). Afterwards, the brains were rinsed 5 times in PBS-TrX for 10 min and 2 times in 0,1 M TRIS buffer (pH 8,5; Tris-Base, HCL) and the incorporated EdU was detected using copper-catalysed click-chemistry^76^. Therefore, the brains were incubated for 1 to 2 hours at room temperature in constant darkness in freshly prepared reaction solution (4 mM CuSO^4^, 10 μM azide-fluorochrome (see Table 1), and 500 mM ascorbic acid in 0.1 M TRIS buffer pH 8.5). Afterwards, the brains were embedded in a mixture of glycerol and PBS (80% glycerol, 20% PBS) or Mowiol^110^ between two coverslips using a layer of two reinforcing rings as spacers.

**Table 1:** Overview of used antibodies and markers.

Name	Abbreviation	Host species	Dilution	Used to label	Vendor/Donor (catalogue #, batch #, RRID/CAS #)	References/Specificity
4′,6-diamidin-2-phenylindol	DAPI		1:20,000	Cell nuclei (DNA)	Sigma Aldrich, Steinheim, Germany (D9542, n/a, 28718-90-3)	^120^
5-ethynyl-2′-desoxyuridine	EdU		100 µM	newly born cells (newly synthesized DNA)	Thermo Fischer Scientific, Rockford, IL, USA (A10044; 1259424; 61135-33-9)	^75,76^
Alexa Flour 488 coupled phalloidine	Phalloidin		1:200	Ubiquitous neuroanatomy (F-Actin)	Thermo Fischer Scientific, Rockford, IL, USA (A12379; n/a; n/a)	^121^
Alexa Flour 488 Azide	488-azide		1 µM	5-ethynyl-2′-desoxuridine	Thermo Fischer Scientific, Rockford, IL, USA (A10260; 1320994; n/a)	
Anti-*Drosophila melanogaster* reversed polarity (4α3)	anti-repo	Rabbit	1:5,000	Glia cells	B. Altenhain, University of Mainz, Germany (n/a; n/a; n/a)	^78,122^
Anti-Red fluorescent protein	RFP	Chicken	1:3,000	DsRed	Rockland Immunochemicals INC, Limerick, PA, USA (600-901-379, 26274, AB_10704808)	
Cy3 coupled goat anti-chicken	GACh-Cy3	Goat	1:300	Primary antibodies raised in chicken	Jackson ImmunoResearch; Westgrove, PA, USA (103-165-155, 93117, AB_2337386)	
Cy3 coupled goat anti-rabbit	GAR-Cy3	Goat	1:300	Primary antibodies raised in rabbit	Jackson ImmunoResearch; Westgrove, PA, USA (111-165-144, n/a, AB_2338006)	
Cy5 Azide	Cy5-azide		1 µM	5-ethynyl-2′-desoxuridine	Jena Bioscience, Jena, Germany (CLK-CCA-9295-1; Kli008-078; n/a)	
Cy5-Sulfo Azide			1 µM	5-ethynyl-2′-desoxuridine	Jena Bioscience, Jena, Germany (CLK-AZ118-1; Kli009-030; n/a)	
Moth-R2, Orco antiserum	Moth-R2	Rabbit	1:5,000	Olfactory sensory neurons	J. Krieger, University Halle-Wittenberg, Germany (n/a; n/a; n/a)	^26^

### Olfactory stimulation

For enhanced olfactory stimulation we reared 20 freshly eclosed adults in mixed-sex groups in para-film sealed 68 ml *Drosophila* vials on about 20 g substrate, supplemented with 1 µl pure green-leaf alcohol (cis-3-hexen-1-ol; Sigma-Aldrich) or 1 µl of the beetles aggregation pheromone 4,8-dimethyldecanal^79^ in a dilution of 1:1000 in silicone oil (4,8-dimethyldecanal; Trécé Inc., Adair, OK, USA; Silicone oil M 10, Carl Roth, Karlsruhe, Germany), on filter paper (1 cm²).

### Orco-knockdown

*Orco* dsRNA (dsRNA^*orco*^) and *DsRed* dsRNA (dsRNA^*sham*^) used as a control were synthesized as previously published^26^. Both dsRNAs were injected into pupae at about 30%–40% of total metamorphosis. The Orco knockdown was verified by immunohistochemistry against Orco (Moth-R2, kindly provided by J. Krieger, University of Hohenheim, Germany) on cryo-sections of antennae as previously published^26^ (Supplemental Fig. S4).

### Image acquisition and processing

Fluorescent preparations were imaged using a widefield microscope setup (Axio Observer Z1; Carl Zeiss Microscopy, Jena, Germany) and a confocal laser scanning microscope (TCS SP5, Leica Microsystems, Wetzlar, Germany). Confocal image stacks were analysed with Amira 6 graphics software (FEI Visualization Sciences Group, Mérignac Cedex, France).

For further processing, snapshots of single sections and projections generated in Amira were processed (global level adjustments, contrast and brightness optimization) in Adobe Photoshop CC (Adobe Systems, San Jose, CA, USA). Final figure arrangements were performed in Adobe Illustrator CC.

### EAG recordings

For electronantennographical recordings, we followed a previously published protocol^80^. The tests were performed using five female beetles aged seven days after dsRNA injection, corresponding to about A4, with three repeated measurements per animal. The antennal response was recorded at 25 Hz via a custom-build amplifier attached to a data acquisition controller (IDAC-4 A/D converter, Syntech, Hilversum, The Netherlands) using EAG 2000 software (Syntech). During the EAG recordings, the antennae were exposed to a constant flow (~3 l/min) of filtered and humidified air. The green leaf volatile cis-3-hexenol and the beetle’s aggregation pheromone DMD^111^ were used as test odours. They were presented as 1 s pulse via a stimulus controller (CS-02, Syntech) that looped in the odour diluted in silicone oil from a filter paper impregnated with 20 µl odour sample. Measurements were performed in minute-long intervals with increasing concentrations (10^−8^–10^−2^). Each repeat was preceded by the measurement of the reaction to DMD (positive control) and silicone oil, which was used as solvent)

### Data analysis and plotting

Analysis of cell numbers and EAG responses including statistical comparison and plotting was performed using Python (version 3.7.3, Python Software Foundation, www.python.org) based custom scripts. Those scripts utilize the SciPy ecosystems (www.scipy.org) modules SciPy (version 1.2.1)^112^, Numpy (version 1.16.2)^113^, Matplotlib (version 3.0.3)^114^, and Pandas (version 0.24.2)^115^, as well as the additional modules scikit-posthocs (version 0.5.1, https://github.com/maximtrp/scikit-posthocs)^116^, Seaborn (version 0.9.0)^117^, and localreg (version 0.2.1, https://github.com/sigvaldm/localreg).

To test for significant differences between experimental groups, Scheirer-Ray-Hare^118^ test (https://github.com/jpinzonc/Scheirer-Ray-Hare-Test), Kruskal-Wallis test from SciPy and posthoc analysis by Dunn’s multiple comparison from scikit-posthocs with p-value correction using the Holm method^119^ were used.

Raw EAG voltage trains exported from EAG 2000 were initially smoothed using robust LOESS (local regression) method from localreg to account for local spikes and subsequently normalized by subtracting the corresponding responses to silicone oil which was used as the solvent to dilute the test odours.

Figures were plotted as vector graphics and final figure arrangements were performed in Adobe Illustrator CC.

### Ethics approval and consent to participate

All experiments involving animals were performed in compliance with the guidelines of the European Union (Directive 2010/63/EU). As all experiments were on insects an approval of the study by an ethics committee was unnecessary.

## Supplementary information

Supplementary information.

Dataset 1.

Dataset 2.

## Data Availability

Except for the original microscope images/stacks, all data generated or analysed during this study and all analysis scripts are included in this published article [and its Supplemental Information]. The confocal image stacks are available from the corresponding author upon request.
