# Supplementary material for: Adult neurogenesis in the mushroom bodies of red flour beetles (Tribolium castaneum, Herbst) is influenced by the olfactory environment
Source: Sci Rep. 2020 Jan 23;10:1090. doi: 10.1038/s41598-020-57639-x (PMC6978414; doi:10.1038/s41598-020-57639-x)
Supplement: Supplementary file 1 — Supplementary information. [file 41598_2020_57639_MOESM1_ESM.docx]

Supplementary information for

**Adult neurogenesis in the mushroom bodies of red flour beetles (*Tribolium castaneum*, Herbst) is influenced by the olfactory environment**

Björn Trebels (1), Stefan Dippel (1), Magdalina Schaaf (1), Karthi Balakrishnan (2), Ernst A Wimmer (3), Joachim Schachtner (1,4,*)

(1) Philipps-University Marburg, Department of Biology, Animal Physiology, Karl-von-Frisch-Str. 8, 35032 Marburg, Germany.

(2) Department of Forest Zoology and Forest Conservation, Georg-August-University Göttingen, Büsgen-Institute, Büsgenweg 3, Göttingen 37077, Germany.

(3) Department of Developmental Biology, Georg-August-University Göttingen, Johann-Friedrich-Blumenbach-Institute for Zoology and Anthropology, GZMB, Ernst-Caspari-Haus, Justus-von-Liebig-Weg 11, Göttingen 37077, Germany.

(4) Clausthal University of Technology, Adolph-Roemer-Str. 2a, 38678 Clausthal-Zellerfeld, Germany

* corresponding author

Table of contents

[Supplementary Figures 2](#_Toc25051728)

[Supplementary Tables 6](#_Toc25051729)

[Supplementary Methods 12](#_Toc25051730)

[Python script for cell number analysis 12](#_Toc25051731)

[Python script for EAG analysis 15](#_Toc25051732)

## Supplementary Figures


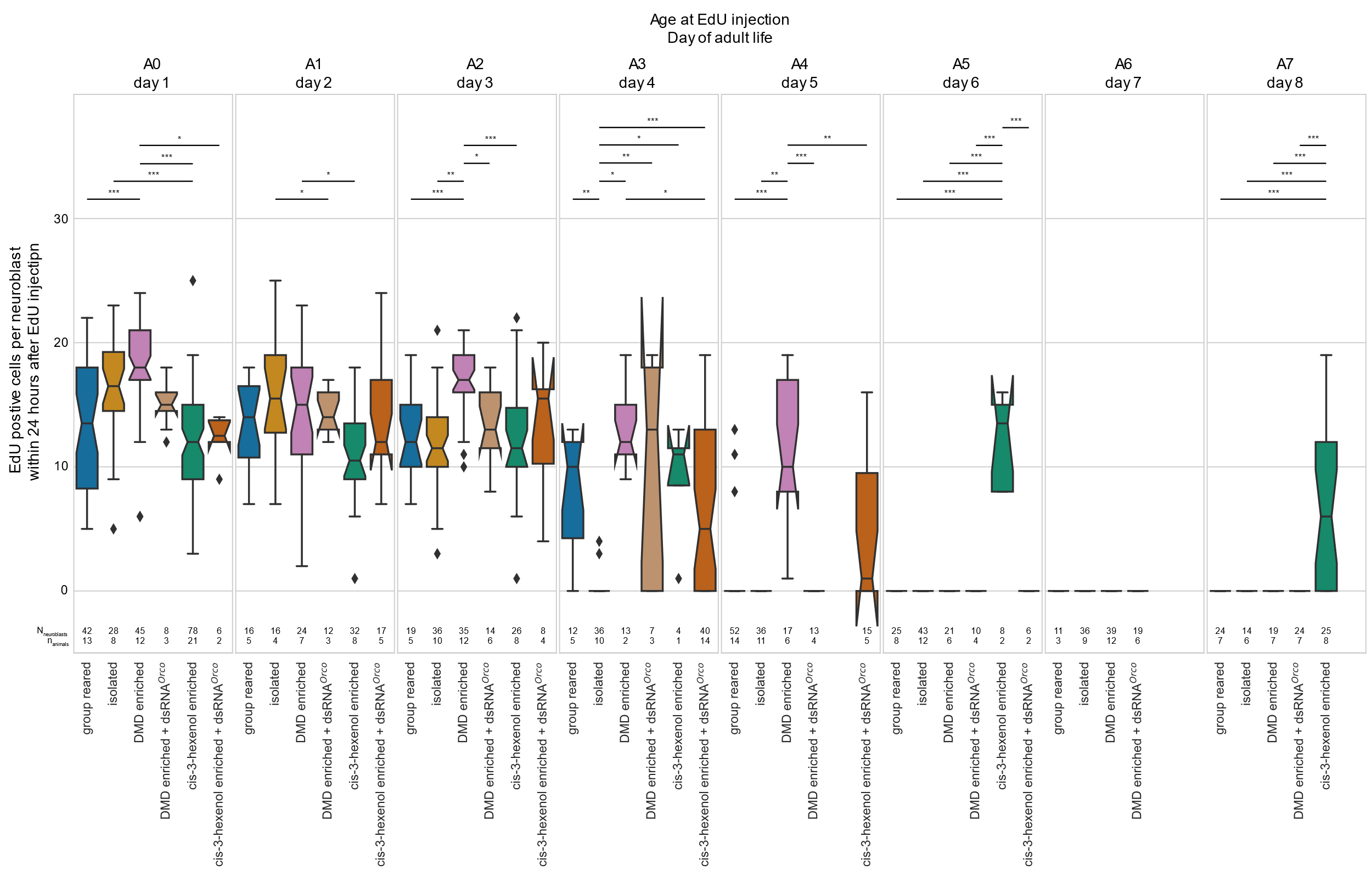


**Supplementary Fig. S1: Number of adult born Kenyon cells under different conditions**

Box plots with whiskers representing the 5-95% percentile of new-born cells per neuroblast within 24 hours after EdU injection during the first week after adult eclosion based on N analysed neuroblasts originating from n beetles. Notches indicate the 95% confidence interval of the median. The bar represents the 25-75 percentile, the line the median, and the diamonds data points outside the 5-95% range. Horizontal lines at 0 indicate presence of neuroblasts, but no occurring neurogenesis. asterisks: statistical significance levels (Holm‑corrected) of difference in median as calculated by Dunn's multiple comparison test (* p_corr_<0.05, ** p_corr_<0.01, *** p_corr_<0.001).


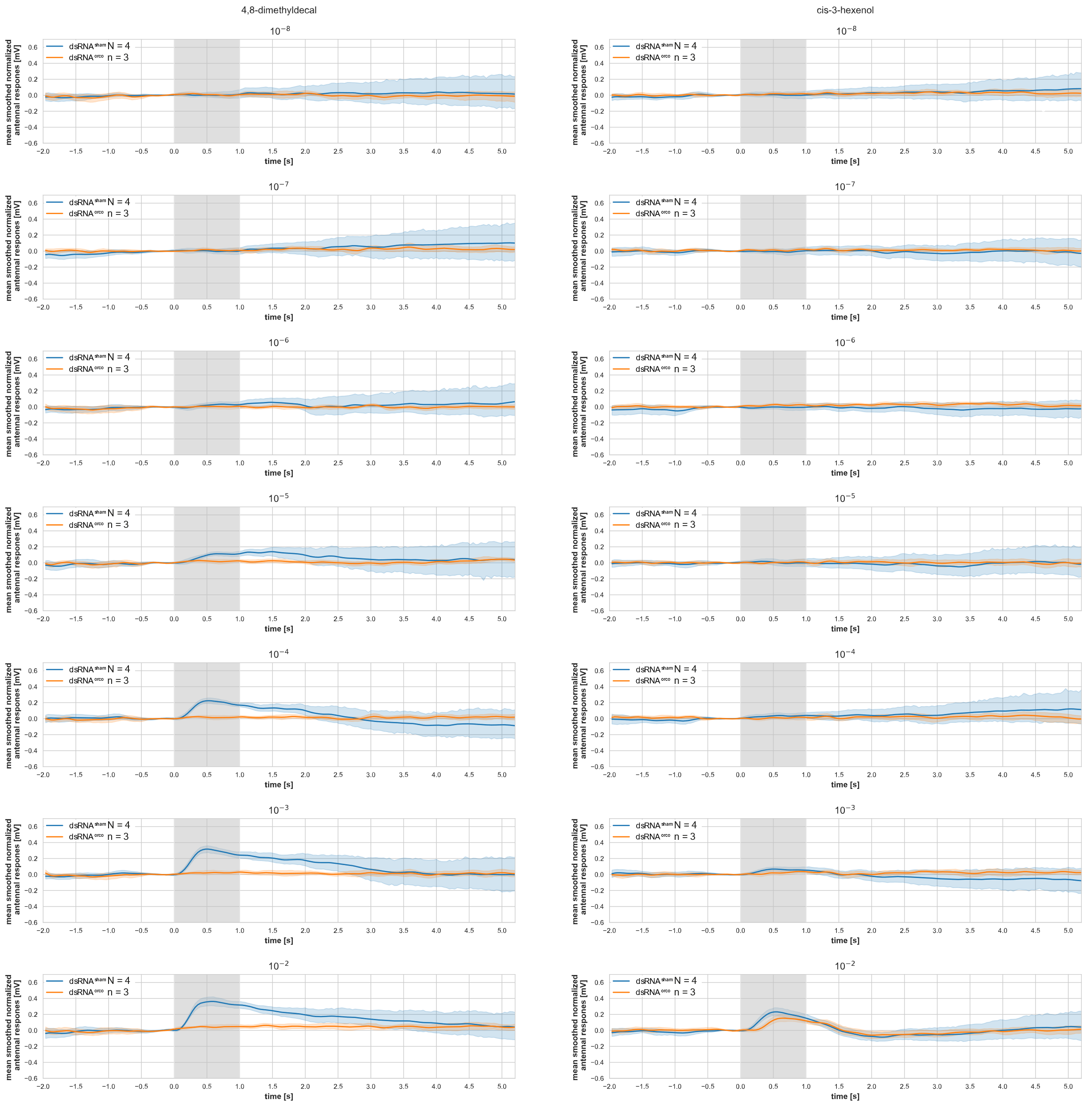


**Supplementary Fig. S2: EAG responses to 4,8-dimethyldecanal and cis‑3‑hexenol**

Line plots of the mean EAG response after robust LOESS smoothing and normilization (substraction of the response to silicone oil, which was used as solvent) to 4,8-dimethyldecanal (DMD) and cis-3-hexenol. Sample sizes are given in the respective subplots. [N] represents the number of animals, while [n] represents the number of replicated per animal. Corespondigly colored shaded areas represent he confidence interval of the mean equivalent to the standard error calculated by bootstrap analysis. Odour stimuli were present for 1s (grey box) and stimulus onset was set to t = 0.


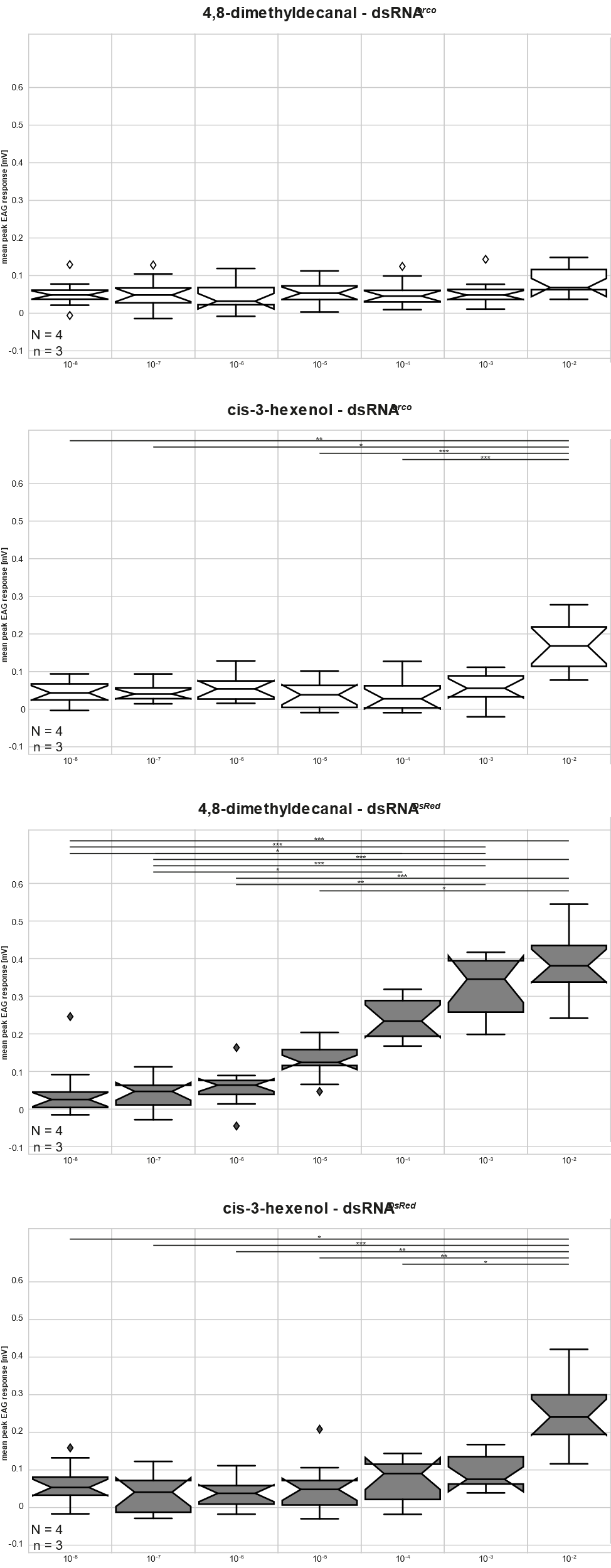


**Supplementary Fig. S3: Peak EAG responses to 4,8-dimethyldecanal and cis‑3‑hexenol**

Box plots with whiskers representing the 5-95% percentile of the peak EAG response after robust LOESS smoothing and normalization (subtraction of the response to silicone oil, which was used as solvent) to 4,8-dimethyldecanal (DMD) and cis-3-hexenol. Sample sizes are given in the respective subplots. [N] represents the number of animals, while [n] represents the number of replicated per animal. Notches indicate the 95% confidence interval of the median. The bar represents the 25-75 percentile, the line represents the median and the diamonds represent data points outside the 5-95% range. Statistical analysis between the different odour dilutions per dsRNA and odour was performed by Kruskal Wallis test followed by posthoc-analysis with Dunn’s multiple comparison test. asterisks: statistical significance levels (Holm‑corrected) of difference in median (* p_corr_<0.05, ** p_corr_<0.01, *** p_corr_<0.001).


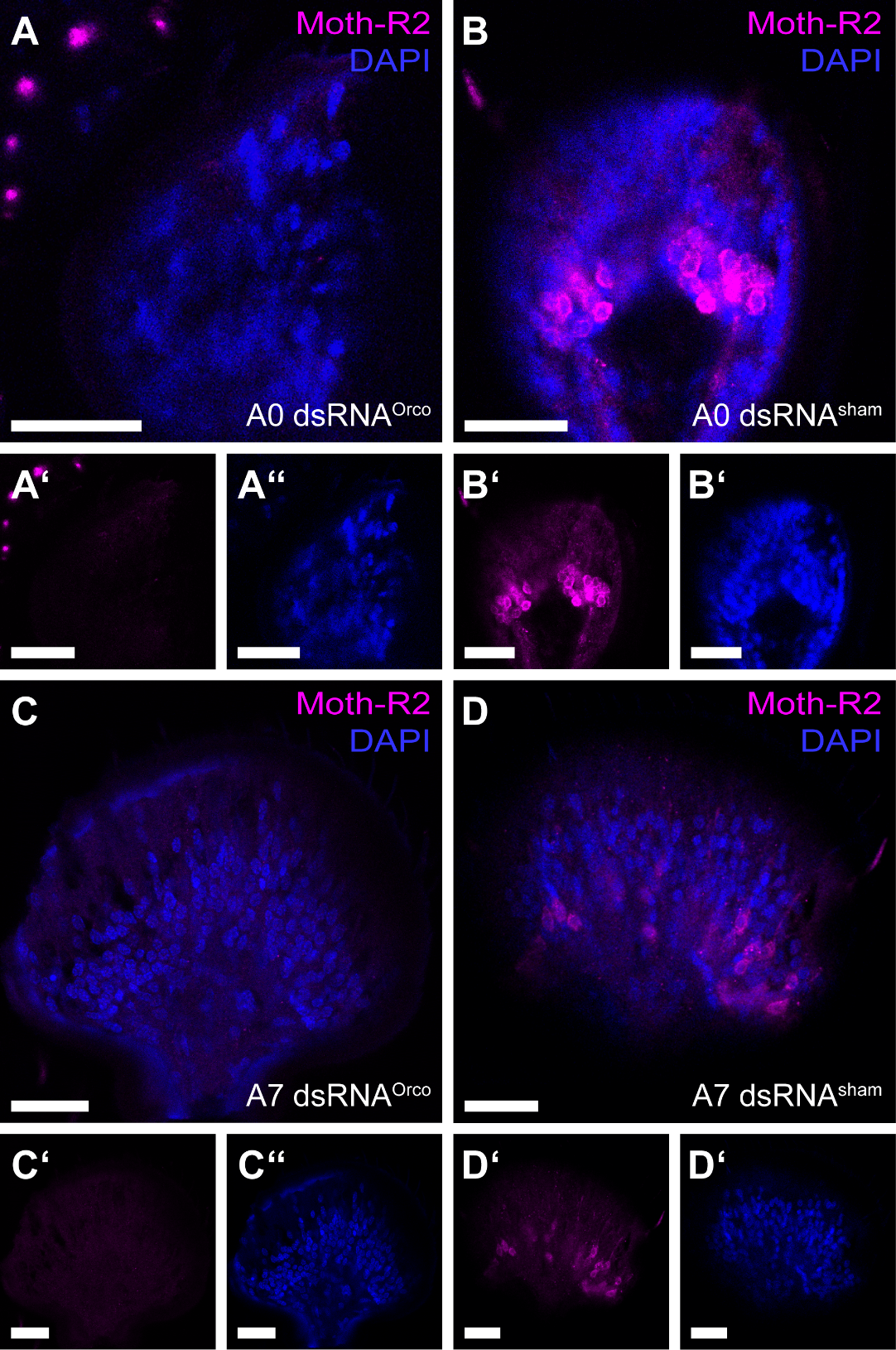


**Supplementary Fig. S4: Verification of the Orco knockdown**

Representative optical sections from antennal cryo-sections of freshly eclosed (A0) and seven days old (A7) beetles of the San Bernadino strain after dsRNA^Orco^ or of the black strain after dsRNA^sham^ treatment respectively. Depicted in blue cell nuclei stained with DAPI and in magenta Orco expressing OSNs with the Moth-R2 antiserum.

There is no detectable staining in the antenna after *Orco* knockdown, whereas in the antenna of the sham treated beetles, the odorant receptor neurons (*OSNs*) are clearly labelled by the antiserum. Scalebars 20 µm.

## Supplementary Tables

**Supplementary Tab. S1: Comparison of cell numbers per age between of both sexes**

The number of neuroblasts “N” originating from “n” animals is given for both sexes for each age of EdU injection (A0 – A7) and experimental groups. Numbers of newborn cells per neuroplasts in males and females were compared by Kruskal-Wallis test. Rounded test results (p-values) are given for each age and experimental group if data from both sexes was obtained.

|  |  | A0  day 1 | A1  day 2 | A2  day 3 | A3  day 4 | A4  day 5 | A5  day 6 | A6  day 7 | A7  day 8 |
| --- | --- | --- | --- | --- | --- | --- | --- | --- | --- |
| group-reared | n_animals-male_ | 7 | 3 | 3 | 3 | 10 | 3 | 2 | 3 |
|  | N_neuroblasts-male_ | 20 | 10 | 11 | 7 | 36 | 10 | 8 | 10 |
|  | n_animals-female_ | 6 | 2 | 2 | 2 | 4 | 5 | 1 | 4 |
|  | N_neuroblasts-female_ | 22 | 6 | 8 | 5 | 16 | 15 | 3 | 14 |
|  | p | 0.3063 | 0.7000 | 0.1038 | 0.8059 | 0.2309 | 1.0000 | 1.0000 | 1.0000 |
| isolated | n_animals-male_ | 5 | 3 | 5 | 6 | 6 | 6 | 5 | 1 |
|  | N_neuroblasts-male_ | 16 | 12 | 17 | 22 | 18 | 21 | 20 | 2 |
|  | n_animals-female_ | 3 | 1 | 5 | 4 | 5 | 6 | 4 | 5 |
|  | N_neuroblasts-female_ | 12 | 4 | 19 | 14 | 18 | 22 | 16 | 12 |
|  | p | 0.4980 | 0.1450 | 0.0004 | 0.2526 | 1.0000 | 1.0000 | 1.0000 | 1.0000 |
| cis-3-hexenol enriched | n_animals-male_ | 9 | 5 | 4 | 1 | 0 | 2 | 0 | 5 |
|  | N_neuroblasts-male_ | 35 | 20 | 13 | 4 | 0 | 8 | 0 | 15 |
|  | n_animals-female_ | 12 | 3 | 4 | 0 | 0 | 0 | 0 | 3 |
|  | N_neuroblasts-female_ | 43 | 12 | 13 | 0 | 0 | 0 | 0 | 10 |
|  | p | 0.4682 | 0.2903 | 0.0925 | # | # | # | # | 0.2701 |
| cis-3-hexenol + dsRNA^Orco^ | n_animals-male_ | 1 | 3 | 2 | 4 | 4 | 1 | 0 | 0 |
|  | N_neuroblasts-male_ | 4 | 12 | 5 | 9 | 11 | 2 | 0 | 0 |
|  | n_animals-female_ | 1 | 2 | 2 | 10 | 1 | 1 | 0 | 0 |
|  | N_neuroblasts-female_ | 2 | 5 | 3 | 31 | 4 | 4 | 0 | 0 |
|  | p | 0.2333 | 0.0801 | 0.3682 | 0.2599 | 0.0024 | 1.000 | # | # |
| 4,8-dimethyldecanal enriched | n_animals-male_ | 5 | 5 | 5 | 4 | 2 | 6 | 8 | 4 |
|  | N_neuroblasts-male_ | 19 | 19 | 16 | 7 | 6 | 21 | 25 | 11 |
|  | n_animals-female_ | 7 | 2 | 7 | 2 | 4 | 0 | 6 | 3 |
|  | N_neuroblasts-female_ | 26 | 5 | 19 | 6 | 11 | 0 | 14 | 8 |
|  | p | 0.0951 | 0.4750 | 0.5694 | 0.0698 | 0.5767 | # | 1.000 | 1.000 |
| 4,8-dimethyldecanal + dsRNA^Orco^ | n_animals-male_ | 0 | 0 | 2 | 0 | 1 | 3 | 3 | 3 |
|  | N_neuroblasts-male_ | 0 | 0 | 5 | 0 | 3 | 6 | 8 | 10 |
|  | n_animals-female_ | 3 | 3 | 4 | 2 | 3 | 1 | 3 | 4 |
|  | N_neuroblasts-female_ | 8 | 12 | 9 | 7 | 10 | 4 | 11 | 14 |
|  | p | # | # | 0.7862 | # | 1.0000 | 1.0000 | 1.0000 | 1.0000 |

**Supplementary Tab. S2: Comparison of the different experimental group within one age**

Numbers of newborn cells per neuroplasts over the different injection times (A0 to A7) within one experimental group were compared by Kruskal‑Wallis test with posthoc analysis using Dunns’ multiple comparison test. Rounded test results (p-values) are given for each age and experimental group if data from both sexes was obtained. The results were used to indicate signifacances in Supplemental Fig. S1.

| **Isolated** |  | **A0**  **day 1** | **A1**  **day 2** | **A2**  **day 3** | **A3**  **day 4** | **A4**  **day 5** | **A5**  **day 6** | **A6**  **day 7** | **A7**  **day 8** |
| --- | --- | --- | --- | --- | --- | --- | --- | --- | --- |
|  | **A0**  **day 1** | -1 | 1 | 1 | 4,84007E-17 | 3,23302E-18 | 1,38068E-19 | 3,23302E-18 | 4,67005E-11 |
|  | **A1**  **day 2** | 1 | -1 | 1 | 3,38402E-11 | 5,72703E-12 | 1,44637E-12 | 5,72703E-12 | 3,18022E-08 |
|  | **A2**  **day 3** | 1 | 1 | -1 | 8,67465E-13 | 7,14879E-14 | 4,55083E-15 | 7,14879E-14 | 5,0367E-08 |
|  | **A3**  **day 4** | 4,84007E-17 | 3,38402E-11 | 8,67465E-13 | -1 | 1 | 1 | 1 | 1 |
|  | **A4**  **day 5** | 3,23302E-18 | 5,72703E-12 | 7,14879E-14 | 1 | -1 | 1 | 1 | 1 |
|  | **A5**  **day 6** | 1,38068E-19 | 1,44637E-12 | 4,55083E-15 | 1 | 1 | -1 | 1 | 1 |
|  | **A6**  **day 7** | 3,23302E-18 | 5,72703E-12 | 7,14879E-14 | 1 | 1 | 1 | -1 | 1 |
|  | **A7**  **day 8** | 4,67005E-11 | 3,18022E-08 | 5,0367E-08 | 1 | 1 | 1 | 1 | -1 |
| **group reared** |  | **A0**  **day 1** | **A1**  **day 2** | **A2**  **day 3** | **A3**  **day 4** | **A4**  **day 5** | **A5**  **day 6** | **A6**  **day 7** | **A7**  **day 8** |
|  | **A0**  **day 1** | -1 | 1 | 1 | 0,612922288 | 8,3619E-18 | 2,47493E-13 | 1,41736E-07 | 5,18338E-13 |
|  | **A1**  **day 2** | 1 | -1 | 1 | 0,754034011 | 4,92846E-10 | 7,15884E-09 | 4,84177E-06 | 9,47272E-09 |
|  | **A2**  **day 3** | 1 | 1 | -1 | 1 | 1,3442E-09 | 2,35011E-08 | 1,68483E-05 | 3,13324E-08 |
|  | **A3**  **day 4** | 0,612922288 | 0,754034011 | 1 | -1 | 0,002167139 | 0,002585399 | 0,020484094 | 0,00266697 |
|  | **A4**  **day 5** | 8,3619E-18 | 4,92846E-10 | 1,3442E-09 | 0,002167139 | -1 | 1 | 1 | 1 |
|  | **A5**  **day 6** | 2,47493E-13 | 7,15884E-09 | 2,35011E-08 | 0,002585399 | 1 | -1 | 1 | 1 |
|  | **A6**  **day 7** | 1,41736E-07 | 4,84177E-06 | 1,68483E-05 | 0,020484094 | 1 | 1 | -1 | 1 |
|  | **A7**  **day 8** | 5,18338E-13 | 9,47272E-09 | 3,13324E-08 | 0,00266697 | 1 | 1 | 1 | -1 |
| **group and 4,8-dimethyldecanal enriched** |  | **A0**  **day 1** | **A1**  **day 2** | **A2**  **day 3** | **A3**  **day 4** | **A4**  **day 5** | **A5**  **day 6** | **A6**  **day 7** | **A7**  **day 8** |
|  | **A0**  **day 1** | -1 | 0,358547436 | 1 | 0,138862716 | 0,01976019 | 4,03047E-14 | 1,65664E-20 | 3,36706E-13 |
|  | **A1**  **day 2** | 0,358547436 | -1 | 1 | 1 | 1 | 2,76835E-06 | 2,69442E-08 | 5,66873E-06 |
|  | **A2**  **day 3** | 1 | 1 | -1 | 0,493493138 | 0,147034108 | 5,6121E-11 | 2,50909E-15 | 2,53006E-10 |
|  | **A3**  **day 4** | 0,138862716 | 1 | 0,493493138 | -1 | 1 | 0,003417715 | 0,000754315 | 0,004009721 |
|  | **A4**  **day 5** | 0,01976019 | 1 | 0,147034108 | 1 | -1 | 0,003568221 | 0,000648657 | 0,004329133 |
|  | **A5**  **day 6** | 4,03047E-14 | 2,76835E-06 | 5,6121E-11 | 0,003417715 | 0,003568221 | -1 | 1 | 1 |
|  | **A6**  **day 7** | 1,65664E-20 | 2,69442E-08 | 2,50909E-15 | 0,000754315 | 0,000648657 | 1 | -1 | 1 |
|  | **A7**  **day 8** | 3,36706E-13 | 5,66873E-06 | 2,53006E-10 | 0,004009721 | 0,004329133 | 1 | 1 | -1 |
| **group and 4,8-dimethyldecanal enriched + dsRNA^Orco^** |  | **A0**  **day 1** | **A1**  **day 2** | **A2**  **day 3** | **A3**  **day 4** | **A4**  **day 5** | **A5**  **day 6** | **A6**  **day 7** | **A7**  **day 8** |
|  | **A0**  **day 1** | -1 | 1 | 1 | 1 | 5,83761E-05 | 0,000160087 | 1,41801E-05 | 6,34005E-06 |
|  | **A1**  **day 2** | 1 | -1 | 1 | 1 | 1,65623E-05 | 7,10422E-05 | 2,03585E-06 | 5,73375E-07 |
|  | **A2**  **day 3** | 1 | 1 | -1 | 1 | 2,01115E-05 | 9,27781E-05 | 2,09858E-06 | 5,59387E-07 |
|  | **A3**  **day 4** | 1 | 1 | 1 | -1 | 0,046742403 | 0,068143552 | 0,027902765 | 0,021774952 |
|  | **A4**  **day 5** | 5,83761E-05 | 1,65623E-05 | 2,01115E-05 | 0,046742403 | -1 | 1 | 1 | 1 |
|  | **A5**  **day 6** | 0,000160087 | 7,10422E-05 | 9,27781E-05 | 0,068143552 | 1 | -1 | 1 | 1 |
|  | **A6**  **day 7** | 1,41801E-05 | 2,03585E-06 | 2,09858E-06 | 0,027902765 | 1 | 1 | -1 | 1 |
|  | **A7** | 6,34005E-06 | 5,73375E-07 | 5,59387E-07 | 0,021774952 | 1 | 1 | 1 | -1 |
| **group cis‑3‑hexenol enriched** |  | **A0**  **day 1** | **A1**  **day 2** | **A2**  **day 3** | **A3**  **day 4** | **A4**  **day 5** | **A5**  **day 6** |  | **A7**  **day 8** |
|  | **A0**  **day 1** | -1 | 1 | 1 | 1 | 1 | 1 |  | 0,001154556 |
|  | **A1**  **day 2** | 1 | -1 | 1 | 1 | 1 | 1 |  | 0,052875925 |
|  | **A2**  **day 3** | 1 | 1 | -1 | 1 | 1 | 1 |  | 0,011720653 |
|  | **A3**  **day 4** | 1 | 1 | 1 | -1 | 1 | 1 |  | 1 |
|  | **A4**  **day 5** | 1 | 1 | 1 | 1 | -1 | 1 |  | 1 |
|  | **A5**  **day 6** | 1 | 1 | 1 | 1 | 1 | -1 |  | 0,19770883 |
|  |  |  |  |  |  |  |  |  |  |
|  | **A7**  **day 8** | 0,001154556 | 0,052875925 | 0,011720653 | 1 | 1 | 0,19770883 |  | -1 |
| **group and cis‑3‑hexenol enriched + dsRNA^Orco^** |  | **A0**  **day 1** | **A1**  **day 2** | **A2**  **day 3** | **A3**  **day 4** | **A4**  **day 5** | **A5**  **day 6** |  |  |
|  | **A0**  **day 1** | -1 | 1 | 1 | 0,320239746 | 0,20983734 | 0,016002824 |  |  |
|  | **A1**  **day 2** | 1 | -1 | 1 | 0,004877305 | 0,006037926 | 0,000309038 |  |  |
|  | **A2**  **day 3** | 1 | 1 | -1 | 0,047997981 | 0,034154525 | 0,001755258 |  |  |
|  | **A3**  **day 4** | 0,320239746 | 0,004877305 | 0,047997981 | -1 | 1 | 0,187388262 |  |  |
|  | **A4**  **day 5** | 0,20983734 | 0,006037926 | 0,034154525 | 1 | -1 | 0,510586405 |  |  |
|  | **A5**  **day 6** | 0,016002824 | 0,000309038 | 0,001755258 | 0,187388262 | 0,510586405 | -1 |  |  |
|  |  |  |  |  |  |  |  |  |  |
|  |  |  |  |  |  |  |  |  |  |

**Supplementary Tab. S3: Comparison of the different ages within one experimental group**

Numbers of newborn cells per neuroplasts within one experimental group over the different injection times (A0 to A7) were compared by Kruskal‑Wallis test with posthoc analysis using Dunns’ multiple comparison test. Rounded test results (p-values) are given for each age and experimental group if data from both sexes was obtained. The results were used to indicate signifacances in Fig. 2.

| **A0** |  | **group reared** | **isolated** | **group reared and 4,8‑dimethyldecanal enriched** | **group reared and 4,8‑dimethyldecanal enriched + dsRNA^Orco^** | **group reared cis‑3‑hexenol enriched** | **group reared and cis‑3‑hexenol enriched + dsRNA^Orco^** |
| --- | --- | --- | --- | --- | --- | --- | --- |
|  | **group reared** | -1 | 0,117102147 | 0,000138692 | 1 | 0,590386995 | 1 |
|  | **isolated** | 0,117102147 | -1 | 1 | 1 | 1 | 0,316598998 |
|  | **group reared and 4,8‑dimethyldecanal enriched** | 0,000138692 | 1 | -1 | 0,590386995 | 6,36019E-11 | 0,036444773 |
|  | **group reared and 4,8‑dimethyldecanal enriched + dsRNA^Orco^** | 1 | 1 | 0,590386995 | -1 | 0,699818698 | 1 |
|  | **group reared cis‑3‑hexenol enriched** | 0,590386995 | 0,000133143 | 6,36019E-11 | 0,699818698 | -1 | 1 |
|  | **group reared and cis‑3‑hexenol enriched + dsRNA^Orco^** | 1 | 0,316598998 | 0,036444773 | 1 | 1 | -1 |
| **A1** |  | **group reared** | **isolated** | **group reared and 4,8‑dimethyldecanal enriched** | **group reared and 4,8‑dimethyldecanal enriched + dsRNA^Orco^** | **group reared cis‑3‑hexenol enriched** | **group reared and cis‑3‑hexenol enriched + dsRNA^Orco^** |
|  | **group reared** | -1 | 1 | 1 | 1 | 0,841516063 | 1 |
|  | **isolated** | 1 | -1 | 1 | 1 | 0,020427712 | 1 |
|  | **group reared and 4,8‑dimethyldecanal enriched** | 1 | 1 | -1 | 1 | 0,023768744 | 1 |
|  | **group reared and 4,8‑dimethyldecanal enriched + dsRNA^Orco^** | 1 | 1 | 1 | -1 | 0,326369485 | 1 |
|  | **group reared cis‑3‑hexenol enriched** | 0,841516063 | 0,020427712 | 0,023768744 | 0,326369485 | -1 | 0,841516063 |
|  | **group reared and cis‑3‑hexenol enriched + dsRNA^Orco^** | 1 | 1 | 1 | 1 | 0,841516063 | -1 |
| **A2** |  | **group reared** | **isolated** | **group reared and 4,8‑dimethyldecanal enriched** | **group reared and 4,8‑dimethyldecanal enriched + dsRNA^Orco^** | **group reared cis‑3‑hexenol enriched** | **group reared and cis‑3‑hexenol enriched + dsRNA^Orco^** |
|  | **group reared** | -1 | 1 | 0,000115535 | 1 | 1 | 1 |
|  | **isolated** | 1 | -1 | 2,23963E-07 | 1 | 1 | 1 |
|  | **group reared and 4,8‑dimethyldecanal enriched** | 0,000115535 | 2,23963E-07 | -1 | 0,042322766 | 2,62433E-05 | 0,334101551 |
|  | **group reared and 4,8‑dimethyldecanal enriched + dsRNA^Orco^** | 1 | 1 | 0,042322766 | -1 | 1 | 1 |
|  | **group reared cis‑3‑hexenol enriched** | 1 | 1 | 2,62433E-05 | 1 | -1 | 1 |
|  | **group reared and cis‑3‑hexenol enriched + dsRNA^Orco^** | 1 | 1 | 0,334101551 | 1 | 1 | -1 |
| **A3** |  | **group reared** | **isolated** | **group reared and 4,8‑dimethyldecanal enriched** | **group reared and 4,8‑dimethyldecanal enriched + dsRNA^Orco^** | **group reared cis‑3‑hexenol enriched** | **group reared and cis‑3‑hexenol enriched + dsRNA^Orco^** |
|  | **group reared** | -1 | 0,00113321 | 0,650642434 | 1 | 1 | 1 |
|  | **isolated** | 0,00113321 | -1 | 5,56907E-09 | 0,008941583 | 0,031009534 | 4,1284E-05 |
|  | **group reared and 4,8-dimethyldecanal enriched** | 0,650642434 | 5,56907E-09 | -1 | 1 | 1 | 0,031009534 |
|  | **group reared and 4,8-dimethyldecanal enriched + dsRNA^Orco^** | 1 | 0,008941583 | 1 | -1 | 1 | 1 |
|  | **group reared cis‑3‑hexenol enriched** | 1 | 0,031009534 | 1 | 1 | -1 | 1 |
|  | **group reared and cis‑3‑hexenol enriched + dsRNA^Orco^** | 1 | 4,1284E-05 | 0,031009534 | 1 | 1 | -1 |
| **A4** |  | **group reared** | **isolated** | **group reared and 4,8‑dimethyldecanal enriched** | **group reared and 4,8‑dimethyldecanal enriched + dsRNA^Orco^** |  | **group reared and cis‑3‑hexenol enriched + dsRNA^Orco^** |
|  | **group reared** | -1 | 1 | 8,43512E-16 | 1 |  | 0,000970773 |
|  | **isolated** | 1 | -1 | 5,94432E-16 | 1 |  | 0,000355396 |
|  | **group reared and 4,8-dimethyldecanal enriched** | 8,43512E-16 | 5,94432E-16 | -1 | 1,88049E-10 |  | 0,002964403 |
|  | **group reared and 4,8-dimethyldecanal enriched + dsRNA^Orco^** | 1 | 1 | 1,88049E-10 | -1 |  | 0,004061159 |
|  |  |  |  |  |  |  |  |
|  | **group reared and cis‑3‑hexenol enriched + dsRNA^Orco^** | 0,000970773 | 0,000355396 | 0,002964403 | 0,004061159 |  | -1 |
| **A5** |  | **group reared** | **isolated** | **group reared and 4,8‑dimethyldecanal enriched** | **group reared and 4,8‑dimethyldecanal enriched + dsRNA^Orco^** | **group reared cis‑3‑hexenol enriched** | **group reared and cis‑3‑hexenol enriched + dsRNA^Orco^** |
|  | **group reared** | -1 | 1 | 1 | 1 | 1,85387E-20 | 1 |
|  | **isolated** | 1 | -1 | 1 | 1 | 1,09533E-22 | 1 |
|  | **group reared and 4,8-dimethyldecanal enriched** | 1 | 1 | -1 | 1 | 1,31589E-19 | 1 |
|  | **group reared and 4,8-dimethyldecanal enriched + dsRNA^Orco^** | 1 | 1 | 1 | -1 | 3,51266E-15 | 1 |
|  | **group reared cis‑3‑hexenol enriched** | 1,85387E-20 | 1,09533E-22 | 1,31589E-19 | 1,31589E-19 | -1 | 7,59479E-12 |
|  | **group reared and cis‑3‑hexenol enriched + dsRNA^Orco^** | 1 | 1 | 1 | 1 | 7,59479E-12 | -1 |
|  |  |  |  |  |  |  |  |
|  |  |  |  |  |  |  |  |
|  |  |  |  |  |  |  |  |
|  |  |  |  |  |  |  |  |
|  |  |  |  |  |  |  |  |
|  |  |  |  |  |  |  |  |
|  |  |  |  |  |  |  |  |
|  |  |  |  |  |  |  |  |
|  |  |  |  |  |  |  |  |
|  |  |  |  |  |  |  |  |
|  |  |  |  |  |  |  |  |
| **A6** |  | **group reared** | **isolated** | **group reared and 4,8‑dimethyldecanal enriched** | **group reared and 4,8‑dimethyldecanal enriched + dsRNA^Orco^** |  |  |
|  | **group reared** | -1 | 1 | 1 | 1 |  |  |
|  | **isolated** | 1 | -1 | 1 | 1 |  |  |
|  | **group reared and 4,8-dimethyldecanal enriched** | 1 | 1 | -1 | 1 |  |  |
|  | **group reared and 4,8-dimethyldecanal enriched + dsRNA^Orco^** | 1 | 1 | 1 | -1 |  |  |
|  |  |  |  |  |  |  |  |
|  |  |  |  |  |  |  |  |
| **A7** |  | **group reared** | **isolated** | **group reared and 4,8‑dimethyldecanal enriched** | **group reared and 4,8‑dimethyldecanal enriched + dsRNA^Orco^** | **group reared cis‑3‑hexenol enriched** |  |
|  | **group reared** | -1 |  |  |  | 9,38185E-08 |  |
|  | **isolated** |  | -1 |  |  | 6,20432E-06 |  |
|  | **group reared and 4,8-dimethyldecanal enriched** |  |  | -1 |  | 5,60453E-07 |  |
|  | **group reared and 4,8-dimethyldecanal enriched + dsRNA^Orco^** |  |  |  | -1 | 9,38185E-08 |  |
|  | **group reared cis‑3‑hexenol enriched** | 9,38185E-08 | 6,20432E-06 | 5,60453E-07 | 9,38185E-08 | -1 |  |
|  |  |  |  |  |  |  |  |

## Supplementary Methods

### Supplemetary method S1: Python script for cell number analysis

#load modules

import pandas as pd

import matplotlib.pyplot as plt

import seaborn as sns

import scipy.stats as ss

import scikit_posthocs as sp

#global variables

groups = ['group reared', 'isolated', '4,8-dimethyldecanal enriched', '4,8-dimethyldecanal enriched + dsRNA[Orco]', 'cis-3-hexenol enriched', 'cis-3-hexenol enriched + dsRNA[Orco]']

group_labels = ['group reared', 'isolated', 'DMD enriched', 'DMD enriched + dsRNA$^{Orco}$', 'cis-3-hexenol enriched', 'cis-3-hexenol enriched + dsRNA$^{Orco}$']

ages = ['A0', 'A1', 'A2', 'A3', 'A4', 'A5', 'A6', 'A7']

age_labels = ['A0\nday 1', 'A1\nday 2', 'A2\nday 3', 'A3\nday 4', 'A4\nday 5', 'A5\nday 6', 'A6\nday 7', 'A7\nday 8']

sexes = ['male', 'female']

#open datafile

print('-------------------')

print('Loading data')

data = pd.read_excel('data.xlsx', na_values=['NA'])

#filter rows with missing/unusable neuroblasts

data = data[data['NB_state']=='present'].copy()

#separate experimental groups

data_groups = {}

for i in groups:

data_groups[i] = data[data['group']==i].copy()

print('Done')

print('-------------------')

#get oberservations

print('-------------------')

print('get observations')

for i in groups:

data_l = data_groups[i].copy()

N_animals = data_l.groupby('age')['specimen'].nunique()

n_neuroblasts = data_l.groupby('age')['specimen'].count()

output_as = "statistics/observations_" + i + '.xlsx'

with pd.ExcelWriter(output_as) as writer:

N_animals.to_excel(writer, sheet_name='animals')

n_neuroblasts.to_excel(writer, sheet_name='neuroblasts')

print('Done')

print('-------------------')

#do statistics

print('-------------------')

print('Performing statistical tests')

# per group

print('- per group')

for i in data_groups:

#select data

data_group = data_groups[i]

#do statistics

data_kw = [data_group.loc[ids, 'cells'].values for ids in data_group.groupby('age').groups.values()]

check = data_group.cells.nunique()

if check > 1:

H, p = ss.kruskal(*data_kw)

outputkw = pd.DataFrame(data={'type': ['test statistics (H)', 'p-Value'], 'value': [H, p]})

if p < 0.05:

output = sp.posthoc_dunn(data_group, val_col='cells', group_col='age', p_adjust = 'holm')

output_as = "statistics/per_group_" + i + '.xlsx'

with pd.ExcelWriter(output_as) as writer:

outputkw.to_excel(writer, sheet_name='Kruskals Walis')

if p < 0.05:

output.to_excel(writer, sheet_name='Dunn\'s Posthoc')

#do statistics per age

print('- per age')

for i in range(len(ages)):

data_ages = data[data['age']==ages[i]]

#do statistics

data_kw = [data_ages.loc[ids, 'cells'].values for ids in data_ages.groupby('group').groups.values()]

check = data_ages.cells.nunique()

if check > 1:

H, p = ss.kruskal(*data_kw)

outputkw = pd.DataFrame(data={'type': ['test statistics (H)', 'p-Value'], 'value': [H, p]})

if p < 0.05:

output = sp.posthoc_dunn(data_ages, val_col='cells', group_col='group', p_adjust = 'holm')

output_as = "statistics/per_age_" + ages[i] + '.xlsx'

with pd.ExcelWriter(output_as) as writer:

outputkw.to_excel(writer, sheet_name='Kruskals Walis')

if p < 0.05:

output.to_excel(writer, sheet_name='Dunn\'s Posthoc')

#overall

print('- overall')

data_combined = data.copy()

data_combined['all_stat_groups'] = data_combined['age'] + '_' + data_combined['group']

#do statistics

data_kw = [data_combined.loc[ids, 'cells'].values for ids in data_combined.groupby('all_stat_groups').groups.values()]

check = data_combined.cells.nunique()

if check > 1:

H, p = ss.kruskal(*data_kw)

outputkw = pd.DataFrame(data={'type': ['test statistics (H)', 'p-Value'], 'value': [H, p]})

if p < 0.05:

output = sp.posthoc_dunn(data_combined, val_col='cells', group_col='all_stat_groups', p_adjust = 'holm')

output_as = "statistics/overall.xlsx"

with pd.ExcelWriter(output_as) as writer:

outputkw.to_excel(writer, sheet_name='Kruskals Walis')

if p < 0.05:

output.to_excel(writer, sheet_name='Dunn\'s Posthoc')

print('Done')

print('-------------------')

print('-------------------')

print('Start plotting data')

#make pointplot

print('- pointplot')

sns.set_style('whitegrid')

fig, axes = plt.subplots(6,1, figsize=(10,15), dpi=300, sharex=False, sharey=True)

plt.subplots_adjust(left = 0.08 , right = 0.99, bottom = 0.05, top = 0.95, hspace=0.55, wspace = 0)

for i in range(len(groups)):

data_group_name = groups[i]

print(' --' + data_group_name)

data_group = data_groups[data_group_name].copy()

sns.pointplot(data=data_group, x='age', y='cells', join=False, linestyles=["--"], capsize=.1, errwidth=.5, scale = 0.9, color="black", ax=axes[i])

axes[i].set_title(group_labels[i], weight='bold', size='larger')

fig.savefig('figures/pointplot.png')

fig.savefig('figures/pointplot.pdf')

plt.close()

print('- boxplot ages')

my_pal = {'group reared': "#0173b2", 'isolated': "#de8f05", 'cis-3-hexenol enriched': "#029e73", 'cis-3-hexenol enriched + dsRNA[Orco]': "#d55e00", '4,8-dimethyldecanal enriched': "#cc78bc", '4,8-dimethyldecanal enriched + dsRNA[Orco]': "#ca9161"} #colorblind

fig, axes = plt.subplots(1,len(ages), sharey=True, figsize=(15,10), dpi=300)

plt.subplots_adjust(left = 0.05, right = 0.99, bottom = 0.3, top = 0.95, hspace=0.2, wspace = 0.02)

for i in range(len(ages)):

axes[i].set_ylim(-4.99,42.99)

print(' --' + ages[i])

data_ages = data[data['age']==ages[i]]

sns.boxplot(data=data_ages, x='group', y='cells', ax=axes[i], notch=True, palette=my_pal, order=groups)

if i == 0:

axes[i].set_ylabel('adult born Kenyon cells per neuroblast\nwithin 24 hours after EdU injection', weight='bold', size='medium')

else:

axes[i].set_ylabel('')

axes[i].set_title(age_labels[i], weight='bold', size='medium')

xlabels = axes[i].get_xticklabels()

axes[i].set_xticklabels(group_labels, rotation='vertical')

axes[i].set_xlabel('')

fig.savefig('figures/boxplot_ages.png')

fig.savefig('figures/boxplot_ages.pdf')

plt.close()

#Scheirer-Ray-Hare-Test (Implementation from jpinzonc on guithub)

data['rank'] = data.cells.sort_values().rank(numeric_only = float)

rows = data.groupby(['group'], as_index = False).agg({'rank':['count', 'mean', 'var']}).rename(columns={'rank':'row'})

rows.columns = ['_'.join(col) for col in rows.columns]

rows.columns = rows.columns.str.replace(r' _$',"")

rows['row_mean_rows'] = rows.row_mean.mean()

rows['sqdev'] = (rows.row_mean - rows.row_mean_rows)**2

cols = data.groupby(['age'], as_index = False).agg({'rank':['count', 'mean', 'var']}).rename(columns={'rank':'col'})

cols.columns = ['_'.join(col) for col in cols.columns]

cols.columns = cols.columns.str.replace(r' _$',"")

cols['col_mean_cols'] = cols.col_mean.mean()

cols['sqdev'] = (cols.col_mean - cols.col_mean_cols)**2

data_sum = data.groupby(['group', 'age'], as_index = False).agg({'rank':['count', 'mean', 'var']})

data_sum.columns = ['_'.join(col) for col in data_sum.columns]

data_sum.columns = data_sum.columns.str.replace(r' _$',"")

nobs_row = rows.row_count.mean()

nobs_total = rows.row_count.sum()

nobs_col = cols.col_count.mean()

Columns_SS = cols.sqdev.sum()*nobs_col

Rows_SS = rows.sqdev.sum()*nobs_row

Within_SS = data_sum.rank_var.sum()*(data_sum.rank_count.min()-1)

MS = data['rank'].var()

TOTAL_SS = MS * (nobs_total-1)

Inter_SS = TOTAL_SS - Within_SS - Rows_SS - Columns_SS

H_rows = Rows_SS/MS

H_cols = Columns_SS/MS

H_int = Inter_SS/MS

df_rows = len(rows)-1

df_cols = len(cols)-1

df_int = df_rows*df_cols

df_total = len(data)-1

df_within = df_total - df_int - df_cols - df_rows

p_rows = round(1-ss.chi2.cdf(H_rows, df_rows),4)

p_cols = round(1-ss.chi2.cdf(H_cols, df_cols),4)

p_inter = round(1-ss.chi2.cdf(H_int, df_int),4)

results = pd.DataFrame(columns=['var','df','H','p'])

results = results.append([{'var':'Group','df':df_rows,'H':H_rows,'p':p_rows}],ignore_index=True,sort=False)

results = results.append([{'var':'Age','df':df_cols,'H':H_cols,'p':p_cols}],ignore_index=True,sort=False)

results = results.append([{'var':'Group:Age','df':df_int,'H':H_int,'p':p_inter}],ignore_index=True,sort=False)

print(results)

print('Done')

print('-------------------')

### Supplemetary method S2: Python script for EAG analysis

# load modules

import numpy as np

import pandas as pd

import matplotlib.pyplot as plt

import seaborn as sns

import matplotlib

import matplotlib.patches as patches

from matplotlib.ticker import (MultipleLocator, FormatStrFormatter, AutoMinorLocator)

import os

import scipy.stats as ss

import scikit_posthocs as sp

from localreg import *

#import data from structured excel sheets (xlsx only) [mv]:[stimulus][subsessions]; replicates as sheets; one file per animal

#input excel-files must be placed in the folder "excel_data" and have the following filename structure: “dsRNA_odorant_animal” and the sheets must have following names for the 3 replicates: a, b, c (see example.xlsx)

#global variables to be edited to relfect the data

#####################################################################################################

dsRNAs = ['dsRed', 'orco'] #order is also used for boxplplots of peak responses

dsRNA_names = ['dsRNA$^{DsRed}$','dsRNA$^{Orco}$'] # needs ot be in the same order as dsRNAs

odorants = ['dmd', 'hexenol']

animals = ['female_1', 'female_2', 'female_3', 'female_3', 'female_4', 'female_5']

replicates = ['a', 'b', 'c']

subsessions = ['DMD', 'silicone oil','10^-8','10^-7','10^-6','10^-5','10^-4','10^-3','10^-2']

subsessions_to_plot = ['10^-8','10^-7','10^-6','10^-5','10^-4','10^-3','10^-2']

subsessions_to_plot_names = ['10$^{-8}$','10$^{-7}$','10$^{-6}$','10$^{-5}$','10$^{-4}$','10$^{-3}$','10$^{-2}$'] # nice names for plotting, need to be in the same order as subsessions_to_plot

odorant_names = ['4,8-dimethyldecanal', 'cis-3-hexenol'] # needs ot be in the same order as odorants

exclude = ['dsRed_dmd_female_2', 'dsRed_hexenol_female_2', 'orco_dmd_female_1', 'orco_hexenol_female_1'] # enter filename (without extension) of animals datasets to exclude

datapoints_to_use = 300 # equals to sampling frequence x desired time in sec after stimulus onset + 50 <-- 2 seconds before stimulus onset

#do not change from here

#######################################################################################################

#check if all necessary output folders exist and create them if not

if not os.path.exists('csv'):

os.makedirs('csv')

if not os.path.exists('csv_g'):

os.makedirs('csv_g')

if not os.path.exists('figures'):

os.makedirs('figures')

if not os.path.exists('statistics'):

os.makedirs('statistics')

#load data from excel files (filter for files to exclude) into dataframe list and save the subessions to csv

sessions = {}

print('Start importing data from excel')

print('----------------------')

for i in dsRNAs:

for j in odorants:

for k in animals:

for l in replicates:

for m in subsessions:

filename = i + '_' + j + '_' + k

session_name = i + '_' + j + '_' + k + '_' + l + '_' + m

print('Current file is: ' + filename + '.xlsx')

if (filename in exclude):

print('excluded from analysis due to bad raw data')

else:

print('Current sheet is: ' + l)

print('Current test odorant is: ' + j)

print('Current subsession is: ' + m)

print('loading data')

data_in = pd.read_excel('excel_data/' + filename + '.xlsx', index_col=None, na_values=['NA'], sheet_name=l)

data_in.dropna(inplace=True)

data_in = data_in.head(datapoints_to_use)

#select only time, stimulus and subsession column

excol = ['t', 'stimulus', m]

data_in = data_in.filter(items=excol)

data_in.rename(columns = {m:'mV'}, inplace=True)

#add dsRNA, animal, odorant, replicat and subsession

data_in['dsRNA'] = i

data_in['animal'] = k

data_in['odor'] = j

data_in['replicat'] = l

data_in['subsession'] = m

data_in["stimulus"] = pd.to_numeric(data_in["stimulus"], downcast='integer')

#invert mV for nicer display

data_in['mV'] = data_in['mV'] * (-1)

sessions[session_name] = data_in.copy()

print('writing csv')

sessions[session_name].to_csv('csv/' + session_name + '.csv', sep=';', index=None, header=True, decimal='.') #csv with "." as decimal seprator

sessions[session_name].to_csv('csv_g/' + session_name + '.csv', sep=';', index=None, header=True, decimal=',') #csv with "," as decimal seprator

print('done')

print('----------------------')

#create new file for supplements containing everthing

print('combine datasets of the same conditions for supplements')

print('----------------------')

sessions_combined = pd.DataFrame()

for i in sessions:

print('Current session is: ' + i)

sessions_combined = sessions_combined.append(sessions[i])

#save to csv

sessions_combined.to_csv('csv/EAG_data_analyzed_raw.csv', sep=';', index=None, header=True, decimal='.') #csv with "." as decimal seprator

sessions_combined.to_csv('csv_g/EAG_data_analyzed_raw.csv', sep=';', index=None, header=True, decimal=',') #csv with "," as decimal seprator

print('done')

print('----------------------')

#smooth the raw data using robust LOESS method to count for unwanted spikes in the transient voltage train

print('Smooth the data')

print('----------------------')

sessions_smoothed = {}

for i in sessions:

print('Current smoothing: ' + i)

session_l = sessions[i].copy()

x = session_l['t'].to_numpy()

y = session_l['mV'].to_numpy()

smoothed = localreg(x, y, degree=2, kernel=tricube, width=0.3)

session_l['mV'] = smoothed

sessions_smoothed[i] = session_l

print('----------------------')

#normalize the smoothed data by substracting the response to silicone oil

print('Normalize the data')

print('----------------------')

sessions_normalized = {}

for i in dsRNAs:

for j in odorants:

for k in animals:

for l in replicates:

for m in subsessions:

filename = i + '_' + j + '_' + k

if (filename in exclude):

print(filename + '.xlsx was excluded from analysis due to bad raw data')

else:

session_name = i + '_' + j + '_' + k + '_' + l + '_' + m

ref_name = i + '_' + j + '_' + k + '_' + l + '_silicone oil'

print('Current session is: ' + session_name)

session_local = sessions_smoothed[session_name].copy()

session_local_ref = sessions_smoothed[ref_name]

session_local['mV'] = session_local['mV'] - session_local_ref['mV']

sessions_normalized[session_name] = session_local.copy()

print('done')

print('----------------------')

#combine datasets of the same conditions for plotting

print('combine smoothed and nomrlaized datasets of the same conditions')

print('----------------------')

sessions_normalized_combined = pd.DataFrame()

for i in sessions_normalized:

print('Current session is: ' + i)

sessions_normalized_combined = sessions_normalized_combined.append(sessions_normalized[i])

print('done')

print('----------------------')

#plot line graphs with error bars for all desired subsessions

print('plot linegraphs of the mean eag responses over time')

print('----------------------')

fig_rows = len(subsessions_to_plot)

fig_cols = len(odorants)

sns.set_style('white', rc={"lines.linewidth": 0.7})

fig, axes = plt.subplots(fig_rows,fig_cols, figsize=(10,15), dpi=300, sharex=False, sharey=False)

plt.subplots_adjust(top=0.95, bottom=0.05, left=0.1, right=0.95, hspace=0.5, wspace = 0.2)

for i in range(len(odorants)):

data_odorants = sessions_normalized_combined[sessions_normalized_combined['odor']==odorants[i]]

print('Current odor is: ' + odorants[i])

for j in range(len(subsessions_to_plot)):

print('now plotting: ' + subsessions_to_plot[j])

data_plot = data_odorants[data_odorants['subsession']==subsessions_to_plot[j]]

data_plot = data_plot[data_plot['t']<=5]

sns.lineplot(data=data_plot, x='t', y='mV', hue='dsRNA', ax=axes[j,i], palette='colorblind')

#plot.despine()

handles, labels = axes[j,i].get_legend_handles_labels()

axes[j,i].legend(handles=handles[1:], labels=dsRNA_names, columnspacing=1, title="", loc="upper left", ncol=2, frameon=True, fontsize='smaller')

axes[j,i].set_xlim([-2,+5])

axes[j,i].set_ylim([-0.58,0.58])

axes[j,i].set_title(odorant_names[i] + ' ' + subsessions_to_plot_names[j], weight='bold', size='medium')

axes[j,i].set_xlabel('time [s]', weight='bold', size='small')

axes[j,i].set_ylabel('mean EAG response [mV]', weight='bold', size='small')

axes[j,i].tick_params(labelsize='x-small')

axes[j,i].minorticks_on()

# Customize the major grid

axes[j,i].grid(which='major', linestyle='-', linewidth='0.5', color='black')

# Customize the minor grid

axes[j,i].grid(which='minor', linestyle=':', linewidth='0.5', color='grey')

a=[0,-1]

b=[0,1]

c=[1,-1]

d=[1,1]

width = c[0] - a[0]

height = d[1] - a[1]

axes[j,i].add_patch(patches.Rectangle((0, -1), width, height,facecolor="silver",zorder=0))

#save figure as png for quick evaluation and pdf for further external processing

fig.savefig('figures/lineplot.png')

fig.savefig('figures/lineplot.pdf')

plt.close()

print('done')

print('----------------------')

#get the peak responsed during stimulus presentation from the smoothed normlized data

print('get peak responses during stimulus presentation')

print('----------------------')

max_mVs = pd.DataFrame()

for i in sessions_normalized:

data_l = sessions_normalized[i].copy()

#filter values during stimulus presentation

data_l = data_l[data_l['stimulus'] == 1]

peak = data_l.loc[data_l['mV'].idxmax()]

#copy data

max_mVs = max_mVs.append(peak)

#save to csv

max_mVs.to_csv('csv/max_mVs.csv', sep=';', index=None, header=True, decimal='.') #csv with "." as decimal seprator

max_mVs.to_csv('csv_g/max_mVs.csv', sep=';', index=None, header=True, decimal=',') #csv with "," as decimal seprator

print('done')

print('----------------------')

#make boxplots of peak responses

print('plot boxplots of the peak eag respondes during stimulus presentation')

print('----------------------')

sns.set_style('whitegrid')

fig_cols = len(subsessions_to_plot)

fig_rows = len(odorants)

fig, axes = plt.subplots(fig_rows,fig_cols, figsize=(15,10), dpi=300, sharex=False, sharey=True)

plt.subplots_adjust(left = 0.06, right = 0.99, bottom = 0.05, top = 0.9, hspace=0.2, wspace = 0)

for i in range(len(odorants)):

data_odorants = max_mVs[max_mVs['odor']==odorants[i]]

print('Current odor is: ' + odorants[i])

for j in range(len(subsessions_to_plot)):

print('now plotting: ' + subsessions_to_plot[j])

data_plot = data_odorants[data_odorants['subsession']==subsessions_to_plot[j]]

sns.boxplot(data=data_plot, x='dsRNA', y='mV', ax=axes[i,j], notch=True, palette='colorblind', order=dsRNAs)

axes[i,j].set_ylim(-0.12,0.7)

axes[i,j].set_ylabel('')

axes[i,0].set_ylabel('peak EAG response [mV] to \n' + odorant_names[i], weight='bold', size='large')

axes[i,j].set_xticklabels(dsRNA_names)

axes[i,j].set_xlabel('')

axes[i,j].set_title(subsessions_to_plot_names[j], weight='bold', size='large')

#save figure as png for quick evaluation and pdf for further external processing

fig.savefig('figures/boxplot.png')

fig.savefig('figures/boxplot.pdf')

plt.close()

print('done')

print('----------------------')

#compare the peak EAG responses of the dsRNA treatments within the odor/subsession groups

print('compare the peak eag responses during stimulus presentation between dsRNAs for the concentrations')

print('----------------------')

for i in range(len(odorants)):

data_odorants = max_mVs[max_mVs['odor']==odorants[i]]

print('Current odor is: ' + odorants[i])

for j in range(len(subsessions_to_plot)):

print('now comapring: ' + subsessions_to_plot[j])

data_session = data_odorants[data_odorants['subsession']==subsessions_to_plot[j]]

data_kw = [data_session.loc[ids, 'mV'].values for ids in data_session.groupby('dsRNA').groups.values()]

H, p = ss.kruskal(*data_kw)

outputkw = pd.DataFrame(data={'type': ['test statistics (H)', 'p-Value'], 'value': [H, p]})

if p < 0.05:

output = sp.posthoc_dunn(data_session, val_col='mV', group_col='dsRNA', p_adjust = 'holm', sort='true')

with pd.ExcelWriter('statistics/peak_responses_between_dsRNAs_' + odorants[i] + '_' + subsessions_to_plot[j] + '.xlsx') as writer:

outputkw.to_excel(writer, sheet_name='Kruskals Walis')

if p <0.05:

output.to_excel(writer, sheet_name='Dunn\'s Posthoc')

print('done')

print('----------------------')
